# Research Progress on the Role of Vitamin D in Autism Spectrum Disorder

**DOI:** 10.3389/fnbeh.2022.859151

**Published:** 2022-05-10

**Authors:** Jing Wang, Haoyu Huang, Chunming Liu, Yangping Zhang, Wenjuan Wang, Zhuo Zou, Lei Yang, Xuemei He, Jinting Wu, Jing Ma, Yun Liu

**Affiliations:** ^1^Department of Rehabilitation, Kunming Children’s Hospital, Kunming Medical University, Yunnan, China; ^2^Department of Otolaryngology, Head and Neck Surgery, Kunming Children’s Hospital, Kunming Medical University, Yunnan, China

**Keywords:** autism spectrum disorder, vitamin D, neurodevelopmental disorder, pathogenesis, research progress

## Abstract

Autism spectrum disorder (ASD) is a neurodevelopmental disorder that can lead to severe social behavioral difficulties, which mainly manifests as social communication and interaction disorders; narrow interests; and repetitive, stereotyped behaviors. In recent years, the prevalence of ASD has increased annually, and it has evolved from a rare disease to one with a high incidence among childhood developmental disorders. The pathogenesis of ASD is considered to be the interaction of genetic and environmental factors. There is increasing evidence that vitamin D deficiency in pregnancy and early childhood can lead to the occurrence of ASD. Studies have demonstrated that vitamin D intervention can significantly improve the symptoms of ASD, but the underlying mechanism is still unclear. Therefore, exploring the neuroprotective mechanism of vitamin D against ASD is a huge challenge currently being worked on by current basic and clinical researchers, a task which is of great significance for the clinical promotion and optimization of vitamin D in the treatment of ASD. To further clarify the relationship between vitamin D and ASD, this review summarizes the correlation between vitamin D level and ASD, the effects of vitamin D supplementation on ASD, the possible mechanism of vitamin D involved in ASD, and insights from ASD animal models.

## Introduction

Autism spectrum disorder (ASD) is a group of neurodevelopmental disorders characterized by impaired social interaction and communication, repetitive and stereotyped behaviors, limited interests, and abnormalities in sensory processing, generally occurring in early childhood (Lord et al., 2018). The American Psychiatric Association’s *Diagnostic and Statistical Manual of Mental Disorders*, fifth edition, refers to childhood autism, uncategorized generalized developmental disorder, and Asperger’s syndrome collectively as ASD (Battle, 2013). The etiology and pathogenesis of ASD are unknown, although the interaction of genetic and environmental factors is believed to play a role in the occurrence of ASD. The specific pathogenesis still requires further study (Lord et al., 2020). In recent years, it was determined that vitamin D, besides regulating calcium and phosphorus metabolism, also has a significant role in fetal and early postnatal brain development. Mounting evidence suggests that vitamin D likely participates in the pathogenesis of ASD, and vitamin D deficiency may be one of the causes of ASD (Eissa et al., 2018). Meanwhile, some studies have illustrated the fact that vitamin D can improve the core symptoms of ASD in children (Wang et al., 2020). This review discusses the correlation between vitamin D level and ASD, the treatment effects of vitamin D on the symptoms of ASD, the possible mechanism of vitamin D’s involvement in ASD, and the current insights available from ASD animal models.

## Autism Spectrum Disorder

### The Epidemiology of Autism Spectrum Disorder

ASD was first reported in 1943 by Leo Kanner, an American physician, who referred to it as “early infantile autism” (Harris, 2018). ASD was once considered an infrequent disease; nevertheless, the prevalence of ASD has risen dramatically in the last several decades in various countries (Zablotsky et al., 2015). The World Health Organization pointed out that ASD has become one of the most rapidly growing severe diseases in the world, and it is a major public concern that seriously affects the health of children (Lai et al., 2014; Sandin et al., 2017). The U.S. Centers for Disease Control and Prevention reported that the prevalence of autism among 8-year-old children in the United States in 2016 was 1/54, with a 4.3:1 ratio of males to females (Maenner et al., 2020). Surveys in other countries have shown that the prevalence of ASD has increased over time (Lai et al., 2019). Although there are no national investigation data on the epidemic of ASD in China, a multicenter study reported that the prevalence of ASD among those 6–10 years of age is about 1% in China, suggesting a rising tendency (Sun et al., 2019). However, the reason for such a jump has not been fully recognized. ASD is more common in urban than rural areas, and urban areas are associated more closely with cloudy and rainy weather, less ultraviolet B (UVB) exposure, and greater air pollution (Cannell, 2017). These factors reduce ultraviolet radiation at the surface and vitamin D production in the skin, consistent with the etiological hypothesis that vitamin D deficiency might contribute to ASD (Cannell, 2008; Wai et al., 2015).

### The Etiology and Pathogenesis of Autism Spectrum Disorder

The etiology and pathogenesis of ASD are currently unclear. Since the 1980s, research on autism has entered a novel stage, and researchers began to abandon the hypothesis of the cause being so-called “improper parental care.” Medical workers first tried to identify the cause of ASD in the field of biology (Campisi et al., 2018). With the deepening of investigations on ASD, however, we have come to know that ASD is a complex neurodevelopmental disorder caused by the interaction of genetic and environmental factors (Steinman, 2020). Studies have shown that > 1,000 genes are related to ASD. The comorbidity rate of ASD in identical twins is also significantly higher than that in fraternal twins, and some immediate family members of ASD patients have clinical symptoms similar to ASD even if they themselves have not been diagnosed with ASD, such as social and communication disorders, stereotyped behaviors, etc. These findings all suggest that genetic factors play an important role in the pathogenesis of ASD (Famitafreshi and Karimian, 2018; Bölte et al., 2019). However, only 25–30% of ASD children have detectable ASD-related genes, and nearly 70% of cases have a cause that does not involve genetics (Famitafreshi and Karimian, 2018). Therefore, the role of environmental factors in the pathogenesis of ASD cannot be overlooked (Modabbernia et al., 2017; Uçar et al., 2020). Lifestyle and environmental factors, such as nutrition (Bala et al., 2016), medications (Bromley, 2016; Kaplan et al., 2016), toxic substances (Kardas et al., 2016; Skalny et al., 2016), maternal infections during pregnancy (Jiang et al., 2016; Bilbo et al., 2018), stress, and vaccine immunization, have been extensively studied and found to be associated with ASD (Wang et al., 2016). About 1/3 of ASD children have significantly increased serotonin (5-hydroxytryptamine [5-HT]) levels in peripheral blood, and 5-HT reuptake inhibitors can improve the emotional symptoms and repetitive, stereotyped behaviors of ASD patients (Abdulamir et al., 2018). The rise in dopamine levels in the hypothalamus can induce stereotyped behaviors in ASD children, while dopamine-blocking drugs can reduce the stereotyping of ASD children (Kirsten and Bernardi, 2017). Meanwhile, ASD children have abnormal electroencephalographs, brain structure, and brain function (Gibbard et al., 2018). Studies have found that the frontal, parietal, and occipital cortices of ASD patients are thinner. ASD patients also have brain network–connection disorders, and the functional connections between the frontal and temporal cortices of the brain and other brain regions are reduced, resulting in less information transmission (Naaijen et al., 2018; Pagnozzi et al., 2018). Immunological studies have illustrated that the numbers of white blood cells and CD38^+^ B-cells and the levels of HLA-DR (the cell-activation marker of CD8^+^ and CD4^+^ T-cells) are increased, suggesting that there is an imbalance in immunity, in ASD children (Kim et al., 2017). The currently applied maternal immune-activation (MIA) model is a type of ASD model that activates the immune system during pregnancy and leads to disease in the offspring. Pregnant rats were injected with double-stranded polyinosine:cytosine at 9.5 days of gestation, and their offspring showed ASD-like behaviors (Meltzer and Van de Water, 2017). Cannell’s epidemiological survey results revealed that the prevalence of ASD was higher in areas with greater air pollution, urban areas, and those at higher altitudes. These areas have less UVB light, which results in a deficiency of vitamin D. Using these findings, combined with the impact of vitamin D on brain development, the etiological hypothesis that vitamin D deficiency might result in ASD was first proposed (Cannell, 2008). In particular, the factors that cause ASD—such as air pollution, cloudy weather, seasonal factors, migration of dark-skinned immigrants to poleward latitudes, birth order, gestational diabetes, preeclampsia, cesarean delivery, autoimmune disease in the family, and nutrition—are all associated with a deficiency of vitamin D (Alzghoul, 2019).

## Autism Spectrum Disorder and Vitamin D

### Vitamin D

Vitamin D, a fat-soluble vitamin, is a general title for a collection of steroid-like substances, including ergocalciferol and cholecalciferol (D_3_; Grant, 2016). Vitamin D is an essential nutrient for the human body. Vitamin D_3_ is synthesized in the skin by the reaction of 7-dehydrocholesterol with UVB radiation (Bivona et al., 2019). It mainly comes from skin exposure to UVB radiation (Landel et al., 2018). Subsequently, it undergoes 2-step hydroxylation in the liver and kidneys to first form 1,25(OH)D, then 1,25(OH)_2_D_3_, which binds to vitamin D receptors (VDRs) and exerts a biological effect. Because 1,25(OH)_2_D_3_ synthesis is tightly regulated, 25-(OH)D in serum has been suggested to be the best single indicator of vitamin D status. It has been thought that the main role of vitamin D is to regulate calcium and phosphorus metabolism, thus affecting bone growth and development (García-Serna and Morales, 2020). Current studies have found that 1α-hydroxylase, the key enzyme for vitamin D synthesis, and VDRs are widely present in brain tissue, and vitamin D plays a crucial role in brain development (Zhou et al., 2018). Vitamin D has important effects on brain development and function, including neuronal differentiation, proliferation, and apoptosis; regulates synaptic plasticity and the dopaminergic system; and reduces the oxidative burden (Karras et al., 2018). Studies have found that vitamin D_3_ can also promote the development of regulatory T-cells and inhibit an excessive immune response and autoimmune reactions (Mak, 2018). In addition, vitamin D plays an important role in the regulation of gene expression. One study revealed that 223 ASD risk genes in the SFARI database were vitamin D_3_–sensitive genes, which means that these ASD-relevant genes might be regulated by vitamin D (Trifonova et al., 2019).

### The Correlation Between Vitamin D Level and Autism Spectrum Disorder

#### Peripheral Blood Vitamin D Levels in Children With Autism Spectrum Disorder

Since Cannell proposed the hypothesis that vitamin D deficiency may contribute to ASD, an increasing number of researchers have begun to assess changes in serum vitamin D levels among ASD children. A great number of studies investigating the vitamin D status of children and adolescents with ASD from different countries and races reported that ASD children and adolescents had lower vitamin D levels (Fahmy et al., 2016; Basheer et al., 2017; Cieśliñska et al., 2017; Desoky et al., 2017; Garipardic et al., 2017; Altun et al., 2018; Arastoo et al., 2018). Arastoo et al. analyzed the vitamin D levels of 31 ASD children and 31 healthy children and found that 96.8% of the ASD children were deficient in vitamin D (Arastoo et al., 2018). The level of 25-(OH)D in ASD children was significantly lower than that of the control group, and the Social Response Scale (SRS) scores of ASD children with vitamin D deficiency were significantly higher than those of ASD children with normal vitamin D levels (Dong et al., 2017; Guo et al., 2019). Compared to the healthy children, they found that the level of vitamin D in ASD children was lower than that in the control group, and the level of vitamin D was significantly negatively correlated with the total scores of the Autism Behavior Checklist (ABC), Childhood Auditing Scale (CARS), SRS, Autism Treatment Evaluation Checklist (ATEC), behavioral energy zone, and the ATEC social energy zone, indicating that the lower the vitamin D level, the more severe the core symptoms of ASD were. Recently, a meta-analysis of 24 case–control studies demonstrated that children and adolescents with ASD had significantly lower vitamin D concentrations than those of participants in the control group (mean difference, −7.46 ng/mL; 95% confidence interval, −10.26 to −4.66 ng/mL; *p* < 0.0001; I^2^ = 98%; Wang et al., 2020).

#### The Maternal Vitamin D Level During Pregnancy

A higher prenatal 25-(OH)D level exerts a positive influence on the cognitive development of the offspring, and the 25-(OH)D level in early pregnancy may have a stronger influence on the offspring’s neurodevelopment relative to that in the late period (García-Serna and Morales, 2020). Diverse studies have investigated the impact of prenatal exposure to vitamin D on brain development. A systematic review and meta-analysis published in 2019 summarized evidence of the association between 25-(OH)D levels in maternal blood in pregnancy or newborn blood at birth and neurodevelopmental outcomes and found that children with a low prenatal 25-(OH)D (<20 ng/mL) level exhibited more ASD-related symptoms, greater behavioral difficulties, and less social skills at 5 years of age (López-Vicente et al., 2019). In a study of 4,229 children, researchers detected maternal vitamin D levels in the second trimester and at birth, then used SRS to assess ASD-like symptoms in the children until 6 years of age, ultimately finding that vitamin D deficiency during pregnancy increased the risk of ASD-like symptoms in childhood (Vinkhuyzen et al., 2018). Another study demonstrated that mothers in the ASD group had significantly lower maternal serum levels of 25-(OH)D than those in the neurotypical group, with 55.9 and 29.4% of mothers being vitamin D–deficient, respectively (Chen et al., 2016). Lower first-trimester maternal serum levels of 25-(OH)D are associated with a significantly increased risk of ASD development in offspring (*p* < 0.001; Vinkhuyzen et al., 2018). Researchers have also illustrated that decreased vitamin D levels during pregnancy and decreased exposure to solar UVB might increase the risk of ASD (Wang et al., 2016). In 2020, a systematic review found that low vitamin D levels might lead to the development of ASD (Famitafreshi and Karimian, 2018).

#### Vitamin D Level in the Peripheral Blood of Autism Spectrum Disorder Rats

Clinical studies have shown that the use of valproate acid (VPA) during pregnancy is a risk factor for children to suffer from ASD (Wood et al., 2015). The valproic acid (VPA) rat model is a common ASD animal model. The offspring of rats exposed to VPA *in utero* by an intraperitoneal injection of 600 mg/kg of VPA at 12.5 days of gestation showed symptoms consistent with those of ASD children. Researchers tested the level of 25-(OH)D in the peripheral blood of ASD rats treated by VPA at birth and 21 days after birth and recorded a persistent vitamin D deficiency from birth to 21 days after birth, consistent with measurements of the peripheral blood of ASD children (Kim et al., 2013; Selim and Al-Ayadhi, 2013; Ahn et al., 2014). However, this study could not prove that the symptoms of ASD rats were caused by vitamin D deficiency. Vitamin D deficiency in animals causes brain structural and functional alterations similar to those found in humans with ASD. A severe vitamin D deficiency during pregnancy in rats can cause pathological changes, such as brain volume enlargement and ventricular enlargement, and can affect neuron differentiation and axonal connection (Principi and Esposito, 2019). The behaviors of offspring born to vitamin D–deficient animals are similar to those of young children with ASD. An animal model of the development of vitamin D (DVD) deficiency has been proven to reproduce the phenotype related to ASD in the domain of neuroanatomy (Ali et al., 2019). Meanwhile, some studies have reported that vitamin D has preventive or therapeutic effects in ASD rats induced by propionic acid (Alfawaz et al., 2014).

## The Impact of Vitamin D Administration on Symptoms of Autism Spectrum Disorder

### The Preventive Effects of Vitamin D on Autism Spectrum Disorder

A systematic review and meta-analysis published in 2019 summarized evidence of the association between 25-(OH)D levels in maternal blood during pregnancy or newborn blood at birth and neurodevelopmental consequences (García-Serna and Morales, 2020). The meta-analysis offered evidence that prenatal exposure to increased 25-(OH)D levels is associated with improved cognitive development and a reduced risk of attention-deficit/hyperactivity disorder and ASD-related traits later in life (Stubbs et al., 2016). Researchers enrolled mothers who had given birth to ASD children already and provided them with 5,000 IU of supplemental vitamin D daily during subsequent pregnancies. Then, after delivery, each mother was given 7,000 IU of supplemental vitamin D daily during lactation or 1,000 IU of supplemental vitamin D daily if their child was not breastfed until they reached 1 year of age. The study investigators found that the ASD prevalence of these children was reduced to 1/4 (5 vs. 20%) compared to reports in the literature. Nevertheless, some scholars believe that this result needs to be carefully considered because this study was uncontrolled and included an exceedingly low number of pregnant women with varied durations of vitamin D supplementation; they argue that the current data do not support the hypothesis that vitamin D supplementation during pregnancy can prevent the development of ASD (Principi and Esposito, 2019).

### The Therapeutic Effect of Vitamin D on Autism Spectrum Disorder

Quite a few studies have also demonstrated that vitamin D can help to improve symptoms of ASD children. The first report of the use of vitamin D_3_ for ASD treatment dates to 2014 wherein a 32-month-old boy with ASD and vitamin D deficiency was administered 150,000 IU of vitamin D_3_ intramuscularly every month and 400 IU/day orally for 2 months and showed improvements in ASD core symptoms in a transitory amount of time (Jia et al., 2015). In 2015, a randomized controlled trial reported that the CARS scores and social intelligence quotients of ASD children were better than those in the control group after 3 months of supplementation with vitamin D (Azzam et al., 2015). A recent clinical study found that daily high-dose vitamin D (300 U/kg⋅d) supplementation significantly improved the core symptoms of ASD children as mainly reflected in the CARS score, stereotypes, and greater eye contact and attention duration (Saad et al., 2019). During a long-term follow-up study of vitamin D treatment for ASD children, in 37 children with ASD [25-(OH)D < 75 nm/L] who were supplemented with vitamin D for 3 months, ASD symptoms were significantly improved when assessed using the ABC and CARS scores (Feng et al., 2017). One study found that early intervention with vitamin D could improve the growth, development, and behavioral performance of ASD rats, and vitamin D had a therapeutic effect on ASD rats. On the contrary, vitamin D_3_ supplementation was reported to have no effect in a double-blind, randomized, placebo-controlled trial in which 38 children (mean age, 7.1 years) were enrolled (Kerley et al., 2017). Among them, 18 were given vitamin D_3_ (2,000 IU/day for 20 weeks) and 20 received placebo therapy. Serum 25-(OH)D_3_ levels were measured before vitamin D_3_ administration and at the end of the study period, and ASD symptoms were evaluated before and after supplementation by parents and clinicians using the ABC, SRS, and Developmental Disabilities—Children’s Global Assessment Scale (DD-CGAS). Although the treatment group showed a significant increase in serum 25-(OH)D_3_ concentrations (23.4 vs. 34.4 ng/mL) compared to patients receiving placebo therapy (20.7 vs. 20.2 ng/mL; *p* = 0.0016), no improvements in scores of the SRS, DD-CGAS, or 5 ABC subscales were recorded. Similarly, some scholars have found that oral 2,000 U/d of vitamin D_3_ can increase vitamin D levels in children with ASD, but their symptoms of ASD did not improve. Researchers believe that, in order to determine whether ASD symptoms have improved due to vitamin D supplementation given to alleviate insufficient vitamin D levels, it is essential to further increase the vitamin D supplement dose (Principi and Esposito, 2019).

## The Possible Mechanism of Vitamin D Involved in Autism Spectrum Disorder

It has been hypothesized that ASD is a combination of both organ-specific physiologic and systematic abnormalities, such as gene mutations, oxidative stress, an impaired detoxification system, inflammation, immune dysregulation, abnormal neurotrophic factor and neurotransmitter levels, and seizures—at least, in a subset of individuals with ASD (Rossignol and Frye, 2014). Mounting evidence suggests that low vitamin D levels are involved in the etiology of the aforementioned abnormalities (Groves et al., 2014).

### Vitamin D and Gene Mutations

ASD is partly genetically derived (Sandin et al., 2014). A study linking vitamin D metabolic gene variants to ASD risk illustrated that the risk for ASD was increased in children inheriting the AA genotype of the *GC* gene (vitamin D–binding protein), the GG genotype of the *CYP2R1* gene (a catalyst enzyme involved in the transformation of vitamin D to 25-(OH)D), and the paternal Taq l and Bsml genotypes of the *VDR* gene, highlighting the possible etiological role of low vitamin D levels in ASD (Schmidt et al., 2015). Current genetic studies on ASD have found that there are multiple neonatal mutations in affected children (De Rubeis et al., 2014). Vitamin D can perform DNA-repair and -maintenance functions through a variety of mechanisms. At present, it has been confirmed that > 5 vitamin D–dependent genes encode DNA-repair proteins as full-time DNA mutation–repair proteins (Fleet et al., 2012). In addition, studies have found that, when the vitamin D level is reduced, the DNA repair enzyme poly(adenosine diphosphate ribose) polymerase tends to overreact and damage neighboring DNA, and daily supplementation with minor doses of vitamin D_3_ can increase Bax levels, promote apoptosis, and prevent gene mutations (Fedirko et al., 2009). Studies have found that Growth arrest and DNA-damage-inducible alpha, p53, RAD23 homolog B, Proliferating cell nuclear antigen, Poly(adenosine diphosphate ribose) and polymerase Death associated protein 1α (Trifonova et al., 2019), which are closely related to vitamin D, have the function of repairing DNA damage (Fedirko et al., 2009; Fleet et al., 2012; De Rubeis et al., 2014) (Table 1). Therefore, it is speculated that vitamin D deficiencies may cause novel gene mutations in children with ASD, and the multiple novel mutations currently found in children with ASD may be the result of vitamin D deficiencies rather than a pathogenic factor of ASD (Shan et al., 2016; Siracusano et al., 2020).

**TABLE 1:** Vitamin D and gene-mutation repair (Fedirko et al., 2009; Fleet et al., 2012; De Rubeis et al., 2014).

Repair proteins/enzymes	Function
Growth arrest and DNA-damage-inducible alpha	DNA-damage repair
p53	DNA-damage repair
RAD23 homolog B	DNA-damage repair
Proliferating cell nuclear antigen	DNA-damage repair
Death-associated protein 1α (Trifonova et al., 2019)	DNA-damage repair
Poly(adenosine diphosphate ribose) polymerase	DNA-damage repair
Bax	Promoting apoptosis and preventing gene mutations

### Vitamin D Deficiency Leads to Excessive Proliferation of Neuronal Cells

Early brain overgrowth is considered an important neuropathological feature of ASD. Magnetic resonance imaging studies have revealed that the brain volumes of children with ASD are greater than those of normal children. At the cellular level, the expansion of the ASD brain involves quite a few neurons in the anterior prefrontal and dorsolateral prefrontal cortices (Hazlett et al., 2011). Studies have found that vitamin D can inhibit cell proliferation by inducing the proliferation of the cyclin-dependent kinase inhibitors p21 and p27. It can also inhibit cell proliferation by inhibiting the expression of other proteins required for the cell cycle, such as proliferating cell nuclear antigen and cyclin D1 (Marini et al., 2010). An animal model of developmental vitamin D deficiency has been proven to reproduce the phenotype associated with ASD in the field of neuroanatomy (Ali et al., 2018). Therefore, when the vitamin D level is deficient, neuronal cells proliferate excessively, leading to overgrowth of the brain in the early stages of development, which may correlate with the occurrence of ASD.

### Vitamin D and Neurotransmitters

Multiple lines of evidence suggest an involvement of dysregulated neurotransmitter systems (serotonergic, oxytocinergic, and dopaminergic systems) in ASD. These systems play key roles in neurotransmission, brain maturation, cortical organization, and behavior (including social and repetitive behaviors; Staal et al., 2012). Vitamin D-associated neurotransmitters regulates learning, memory, and emotions (Staal et al., 2012; Patrick and Ames, 2014; Pertile et al., 2018) (Table 2). A deficiency of the inhibitory neurotransmitter γ–aminobutyric acid (GABA) in the brain is associated with ASD. Studies have found that long-term treatment of rodents with vitamin D can promote the synthesis of GABA in brain tissues, such as the prefrontal cortex, anterior cingulate cortex, and hippocampus (Staal et al., 2012). Vitamin D influences the synthesis and metabolism of dopamine and the expression of glial cell line–derived neurotropor (GDNF). GDNF is crucial to the survival of dopaminergic neurons, and the absence of vitamin D may be involved in dopamine signal transduction (Pertile et al., 2018). Lower levels of plasma oxytocin and abnormal serotonin concentrations in the brain and tissues outside the blood–brain barrier have been reported in populations with ASD (Patrick and Ames, 2014). While the binding of brain serotonin transporter was significantly lower in high-functioning ASD adults than healthy controls, the binding of brain dopamine transporter was significantly higher in ASD patients (Nakamura et al., 2010). Studies have shown that the concentration of 5-HT in the brains of ASD patients is lower and that of 5-HT in the peripheral blood is higher (Patrick and Ames, 2014). Vitamin D can increase the expression of tyrosine hydroxylase, which is involved in the synthesis of dopamine; vitamin D can also increase the transcription of tryptophan hydroxylase 2, which promotes the synthesis of 5-HT synthetase. 5-HT is a monoamine neurotransmitter, which plays a significant role in regulating emotions in social decision-making. At the same time, a proper amount of vitamin D can inhibit the transcription of tryptophan hydroxylase 1 in peripheral tissues, thus explaining the serotonin paradox in ASD in which peripheral serotonin is increased but central serotonin is decreased (Patrick and Ames, 2014).

**TABLE 2:** Vitamin D–associated neurotransmitters (Staal et al., 2012; Patrick and Ames, 2014; Pertile et al., 2018).

Neurotransmitter	Function
5-hydroxytryptamine	Regulating emotions in social decision-making
Oxytocin	Improving social skills
Seronine	Promote pro-social behavior and assess emotions
Dopamine	Motor control, reward motivation, emotional regulation, and social interaction
γ-aminobutyric acid	Involved in brain cognition, learning, and memory

### The Immunomodulatory Effects of Vitamin D

Studies have demonstrated that immune-activation may be a risk factor for ASD. A lack of vitamin D may alter the immune responses of patients with ASD, and vitamin D may prevent ASD-related behavior dilemmas induced by immune activation (Nakamura et al., 2010). Studies have shown that patients with ASD have higher levels of autoimmune markers, such as anti-nuclear antibodies, anti-ganglioside M1 antibodies, anti-MPB autoantibodies, and anti-nucleosome–specific antibodies. Some studies have shown that the levels of these markers are significantly positively correlated with the severity of ASD (Wang et al., 2016). Vitamin D exerts an immunomodulatory effect through helper T-cells and CD4^+^CD25^+^ regulatory T-cells, and regulatory T-cells prevent autoimmunity by inhibiting Th17 cells (Chambers and Hawrylowicz, 2011). Some scholars have found that vitamin D supplementation can increase the proportion of regulatory T-cells in the body, upregulate the production of dendritic cells, and upregulate interleukin (IL)-10, thereby reducing the intensity of autoimmune attacks, inhibiting damage to tissues by immune cells, and reducing the severity of autoimmune diseases (Saad et al., 2018). Mostafa et al. found that 70% of children with ASD had higher anti-myelin–associated glycoprotein (anti-MAG) levels, and research suggests that serum 25-(OH)D) levels are significantly negatively correlated with anti-MAG levels (Mostafa and Al-Ayadhi, 2012). As the level of anti-MAG correlates with the severity of ASD, this finding suggests that the 25-(OH)D) deficiency in some ASD children is likely a factor that promotes increased anti-MAG levels, and anti-MAG may play a role in brain damage in children with ASD. Vitamin D also modifies the expression of several genes involved in axogenesis and myelination (Ritterhouse et al., 2011). These findings suggest that vitamin D plays a crucial role in auto-antibody production and ASD pathogenesis, perhaps being similar in its manner to other autoimmune diseases like multiple sclerosis and systemic lupus erythematosus (Mazahery et al., 2016).

### The Anti-inflammatory Effects of Vitamin D

Current studies have found that ASD is an inflammation-related disease (Basheer et al., 2017; Cieśliñska et al., 2017). Some studies contend that vitamin D has an immunomodulatory effect, which can enhance the protective immune response and reduce the inflammatory response (El-Sharkawy and Malki, 2020). Vitamin D has different anti-inflammatory effects on the brain, including decreasing harmful inflammatory cytokines and neuro-inflammation caused by oxidants and toxins. ASD individuals have immune function abnormalities similar to those of individuals afflicted by vitamin D deficiency, such as increased inflammatory cytokine levels (Cannell, 2017). Evidence suggests that children with ASD have elevated levels of pro-inflammatory cytokines, including IL-6, tumor necrosis factor alpha (TNF-α), and interferon-γ, in different tissues (Napolioni et al., 2013). When elevated, it is strongly associated with cognitive impairment in ASD (Krakowiak et al., 2017). Vitamin D metabolites have been shown to decrease the secretion of IL-6 and TNF-α, enhance the expression of anti-inflammatory cytokines such as interleukin 10 (IL-10) from activated B-cells, and direct dendritic cells toward a more tolerogenic state. The activation of vitamin D hormone (calcitriol) protects brain tissue by reducing inflammatory cytokine levels (Mazahery et al., 2016).

### The Anti-oxidative Effects of Vitamin D

At present, there are several lines of evidence indicating oxidative stress and mitochondrial dysfunction are prevalent in ASD, and oxidative stress may be a general feature of ASD (Giulivi et al., 2010). Antioxidants, especially glutathione, are the key to early neural survival. Elevated levels of oxidative stress in the brain can damage or interfere with brain development and result in ASD-like symptoms (Jia et al., 2018). It has been found that the concentration of oxidized glutathione in the plasma of ASD children is increased, and the concentration of oxidative stress in these individuals is similarly increased as a result of ASD (James et al., 2006). Vitamin D has an antioxidant effect and can inhibit the synthesis of nitric oxide synthase, upregulate glutathione, reduce glial cell activation and neuro-inflammation, and play a significant role in neuroprotection and neuromodulation (DeLuca, 2016). It has also been reported that vitamin D can directly upregulate certain antioxidant-related genes (such as those that encode superoxide dismutase and thioredoxin reductase; Halicka et al., 2012). Existing evidence also reveals that 25-(OH)D concentrations correlate significantly positively with glutathione levels in healthy adult populations (Alvarez et al., 2014). Therefore, it is believed that vitamin D supplementation can reduce the level of oxidative stress and play a protective role in the brain.

## Insights From Autism Spectrum Disorder Animal Models

Animal models provide advantages over human research due to their controllability, availability, and predictability. They play a crucial role in exploring the etiology and pathogenesis of diseases. The animal model of ASD has become a key platform by which to explore the relationship between vitamin D and ASD. At present, the understanding of ASD and its animal models in medical circles domestic and abroad is extremely limited, and existing ASD animal models may be categorized in three ways: genetic animal models (Südhof, 2017; Dadalko and Travers, 2018; Nakai et al., 2018), VPA-exposure models (Barrett et al., 2017), and MIA models (Kim et al., 2017). Although researchers have reached a consensus on the close relationship between genetic factors and ASD, definite genetic evidence has been found in only 25–30% of ASD cases using existing technical methods. In such cases, the factors at play usually involve chromosome rearrangement and gene copy number variations or point mutation (De Rubeis et al., 2014). The gene models currently in use domestically and abroad include a neuroligins gene model, neurexins gene model, SH3 and multiple ankyrin repeat domains protein 3 gene model, methyl-CpG–binding protein 2 gene model, fragile X mental retardation 1 gene model, and tuberous sclerosis complex 1/2 gene model (Südhof, 2017; Dadalko and Travers, 2018; Nakai et al., 2018). These genes are all found in ASD individuals and have been verified by gene-knockout animal models. Strong evidence of the genetic heritability of ASD is also a key gene for studying gene target regulation. Gene-abnormality models have been created for specific gene deletions and research purposes, and they have specific applications. Regulated environmental factor ASD models include the VPA-exposure model (Barrett et al., 2017), MIA model (Kim et al., 2017), and maternal auto-antibody model (Martínez-Cerdeño et al., 2016). Offspring mice exposed to VPA during pregnancy exhibit typical behavioral performances similar to those of children with ASD, consistent with the current research results of brain structure and function damage and changes in brain transmitters. This model is also the most commonly employed animal model in China (Caspers et al., 2014). To induce the MIA model, pregnant female mice are exposed to polyinosine:cytosine, lipid polysaccharides, simulated viruses, bacterial infections, and other environments to activate the maternal immune system (Boksa, 2010). Studies have found that offspring in the MIA model have behaviors similar to those of children with clinical ASD, which mainly manifest as social impairment and increased repetitive, stereotyped behaviors (Wong and Hoeffer, 2018). At present, several mouse and rat strains have been selected worldwide through behavioral methods that can better simulate the core symptoms of ASD and may be considered to be models of idiopathic ASD; primary examples include the inbred line BTBR-T^+^tf/J mouse model and the inbred line BALB/cByJ mouse model (Chang et al., 2018). Current animal studies of vitamin D and ASD mainly assess the level of vitamin D in the serum and the therapeutic and preventive effects of vitamin D on animal ASD models. Although studies have found lower levels of vitamin D in the peripheral blood of ASD rats, there is currently no animal model of ASD caused by vitamin D deficiency (Kim et al., 2013; Selim and Al-Ayadhi, 2013; Ahn et al., 2014). To further investigate the relationship between vitamin D deficiency and ASD, the behavior of offspring of vitamin D-deficient animals should be studied in the future (Ali et al., 2019). Research has found that serum vitamin D levels of ASD rats were significantly lower than those of normal rats, and the behavior of ASD ratwas aggravated with a reduction in serum vitamin D level in the later developmental stage. At the same time, it was found that a vitamin D intervention could promote the growth and development of ASD rats and improve their ASD-like behaviors. Currently, animals used for vitamin D deficiency disease research include pigs, dogs, rats, and mice. Research on diseases associated with vitamin D deficiency has shifted from rickets to immune, tumor, cardiovascular, and other diseases (Cannell, 2017) and has gradually progressed to cellular and molecular levels. A vitamin D deficiency model constructed by researchers suggests that maternal vitamin D deficiency is one of the important factors that causes embryonic development delay in mice after pregnancy. Nevertheless, whether this embryonic developmental delay can cause ASD in humans remains to be further studied.

## Outlook

The high incidence of ASD has made it a social problem that urgently requires solutions, but the cause of the disease is still unknown. ASD is mostly considered to be the result of a combination of genetic and environmental factors, but current findings concerning the etiology of genetic factors and environmental factors cannot reasonably explain the epidemiological characteristics of ASD, and clinical drug treatments based on various existing pathogenesis have not achieved recognized clinical efficacy. Therefore, it is necessary to explore the etiology and pathogenesis of ASD from a new perspective to provide novel ideas for the treatment of ASD. Low vitamin D levels *in utero*, postnatal, and in early childhood have been hypothesized to be a risk factor for neurodevelopmental disorders, particularly ASD. Animal and human cellular, biological, and physiologic studies have provided compelling evidence for numerous roles of vitamin D in various body processes, some of which are involved in the pathobiology of ASD. Some researchers have found that children with ASD and vitamin D deficiency experience improvements in their core symptoms with an increase in vitamin D levels; however, due to different methods, fewer interventional experiments, and inconsistent results, there is no consensus on the therapeutic effect of vitamin D in ASD, so it is necessary to further implement large-sample, randomized double-blind trials in the future. Progress in understanding the etiology and pathogenesis of vitamin D and ASD requires the conduct of a large number of rigorous scientific experiments. At the same time, we can observe vitamin D levels in ASD animal models and further study the preventive and therapeutic effects and mechanism of vitamin D in this context. If the relationship between vitamin D and ASD is clarified by further research, it would open up a simple, cheap, and safe new path for the prevention and treatment of ASD.

## Author Contributions

JWa, JM, and YL organized this study, reviewed data, and finalized this manuscript. JWa, HH, and ZZ performed the literature search and data analysis. JWu, WW, and CL performed literature management. JWu, YL, XH, and YZ performed literature review and drafted the manuscript. All authors read and approved the final manuscript.

## Conflict of Interest

The authors declare that the research was conducted in the absence of any commercial or financial relationships that could be construed as a potential conflict of interest.

## Publisher’s Note

All claims expressed in this article are solely those of the authors and do not necessarily represent those of their affiliated organizations, or those of the publisher, the editors and the reviewers. Any product that may be evaluated in this article, or claim that may be made by its manufacturer, is not guaranteed or endorsed by the publisher.
